# The Structural Basis of a Prostate Cancer Protein's Unique Selectivity

**DOI:** 10.1371/journal.pbio.0020305

**Published:** 2004-08-24

One of the major players in prostate cancer is a nuclear signaling protein called the androgen receptor. Prostate growth and development is regulated by androgen hormones (like testosterone) that activate the androgen receptor. When an androgen binds the receptor, the receptor binds other proteins, called coactivators, to activate genes controlling cell growth, survival, and differentiation. Unlike other receptors that function in the nucleus, the androgen receptor normally shuns coactivators with a leucine-rich binding domain in favor of those with “aromatic” domains. (Aromatic amino acids are defined by their ring structure.) But during prostate cancer, the receptor interacts with both coactivator types, to promote disease progression.

The secret to a protein's binding preference rests in the underlying sequence of its amino acids, which determines the protein's structure and ultimate behavior. Robert Fletterick and colleagues set out to identify the “full repertoire” of amino acid sequences that might conceivably consort with the androgen receptor. Their findings help explain the unusual behavior of the androgen receptor during prostate cancer progression—a first step toward developing new anticancer therapies.

Treatment for hormone-dependent prostate cancers focuses primarily on reducing androgen levels by using chemicals that compete for androgen receptor docking rights in the hormone-binding pocket of the ligand-binding domain, or LBD. (A ligand is a molecule, like the androgen hormone, that binds to a receptor.) But cancer cells eventually circumvent these chemical assaults through increased levels of either androgen receptors or their coactivators, or through mutations that make androgen receptors immune to chemotherapy. That's why Fletterick and colleagues turned their attentions to the receptor's consorts. Since targeting the hormone-binding pocket of the receptor offers limited benefits, a better strategy might involve disrupting associations with the receptor's coactivators.

Dozens of proteins interact with different regions of the androgen receptor, but the details of these interactions were not known. When a hormone binds to the LBD of other nuclear receptors, it triggers a conformational change that creates a binding surface called AF-2 for the leucine-rich domains of the coactivator proteins. It was not clear, however, how the AF-2 region of the androgen receptor distinguishes between aromatic and leucine-rich domains. To characterize the receptor's binding selectivity, Fletterick and colleagues tested 20 billion peptides, or protein fragments, to see whether they interacted with the LBD region of a hormone-bound androgen receptor.

As expected, most of the peptides that associated with the LBD domain were aromatic. And they interacted with the same region that naturally occurring coactivators bind to. Next, Fletterick and colleagues determined the three-dimensional structure of both the receptor bound to just the androgen hormone and the androgen–receptor pair bound to a subset of seven peptides. The different structures showed that the androgen receptor uses a single surface to bind both leucine-rich and aromatic peptides; when the aromatic peptides have bulky appendages, the receptor's AF-2 domain reorganizes to accommodate them.[Fig pbio-0020305-g001]

**Figure pbio-0020305-g001:**
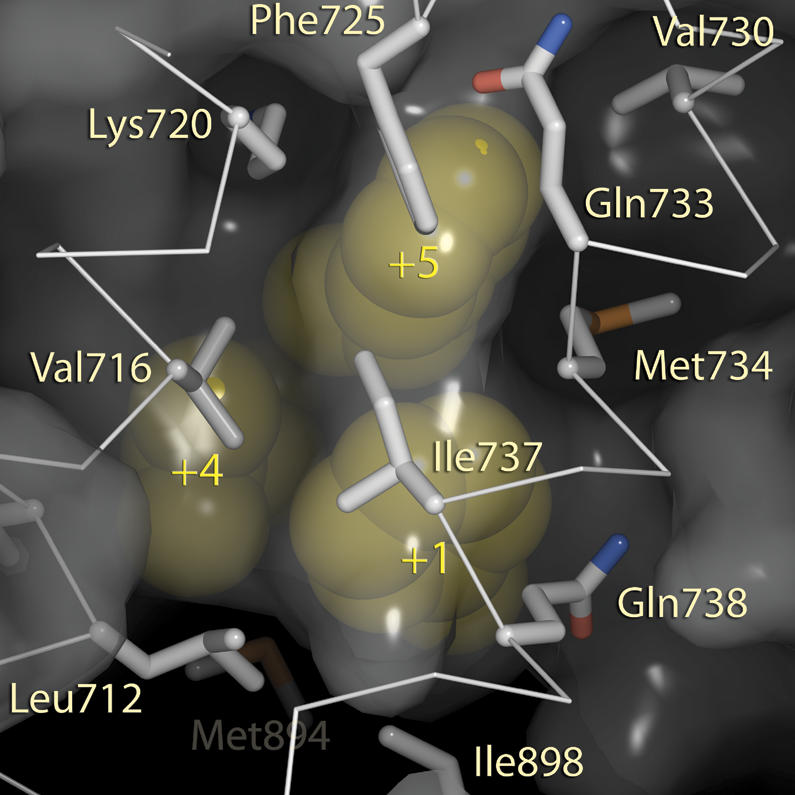
Surface complimentarity of hydrophobic motifs

The various structures and binding affinities for the different receptor–peptide complexes described here show how the receptor can interact with a diverse array of proteins. The androgen receptor, unlike other nuclear receptors, has specific amino acid sequences that better support aromatic peptide binding. Interestingly, mutations in one of these amino acid sequences have been found in prostate cancer. Altogether, the authors conclude, the unique properties of the receptor's AF-2 surface make it “an attractive target for pharmaceutical design.” Drugs that directly interfere with coactivator binding, they explain, are likely to inhibit androgen receptor activity. Here, the authors recommend novel sites on the receptor as promising targets for androgen-receptor-specific inhibitors.

